# Supplementary material for: Thermal tolerance of perovskite quantum dots dependent on A-site cation and surface ligand
Source: Nat Commun. 2023 Apr 18;14:2216. doi: 10.1038/s41467-023-37943-6 (PMC10113222; doi:10.1038/s41467-023-37943-6)
Supplement: Supplementary file 1 — Supplementary Information [file 41467_2023_37943_MOESM1_ESM.pdf]

Supplementary Information for

Thermal tolerance of perovskite quantum dots dependent on A-site cation and surface ligand

Authors: Shuo Wang[1], Qian Zhao[1*] Abhijit Hazarika[2,3], Simiao Li[1], Yue Wu[1], Yaxin Zhai[4], Xihan Chen[5], Joseph M. Luther[3], Guoran Li[1*]

Affiliations:

[1]Institute of New Energy Material Chemistry, School of Materials Science and Engineering, Nankai University, Tianjin 300350, China

[2]Polymers and Functional Materials Division, CSIR-Indian Institute of Chemical Technology, Uppal Road, Tarnaka, Hyderabad 500007, India

[3]National Renewable Energy Laboratory, Golden, CO 80401, USA

[4]Key Laboratory of Low-Dimensional Quantum Structures and Quantum Control of Ministry of Education, Department of Physics, Hunan Normal University, Changsha, Hunan 410081, China

[5]SUSTech Energy Institute for Carbon Neutrality, Department of Mechanical and Energy Engineering, Southern University of Science and Technology, Shenzhen, Guangdong 518055, China

*Correspondence to guoranli@nankai.edu.cn; qian.zhao@nankai.edu.cn

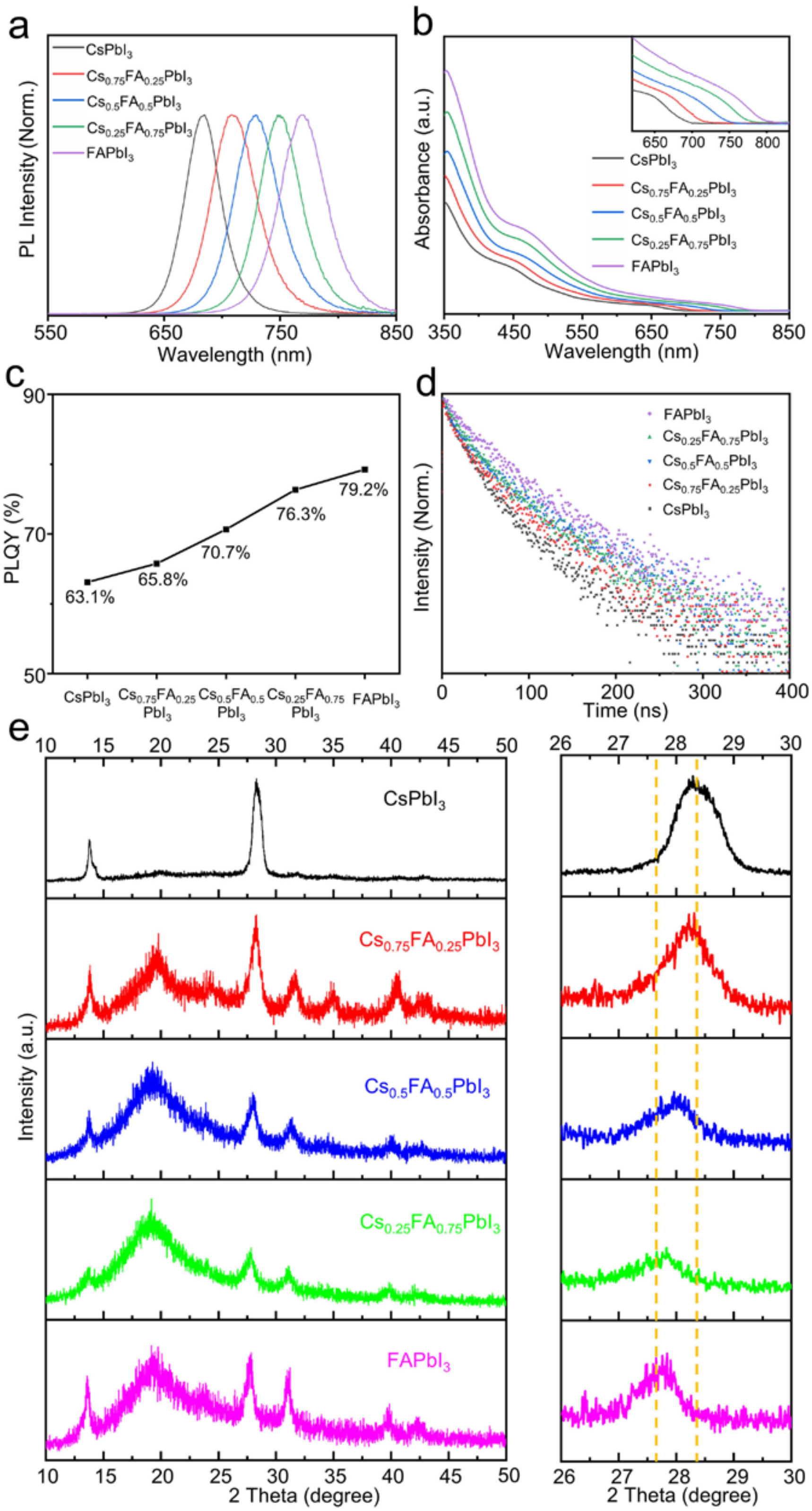

Supplementary Figure 1. Optical and structural properties of as-synthesized $Cs_xFA_{1-x}PbI_3$ QDs. a-e UV-Vis, PL spectra, PLQY, TRPL, and XRD patterns of $Cs_xFA_{1-x}PbI_3$ QDs with different compositions.

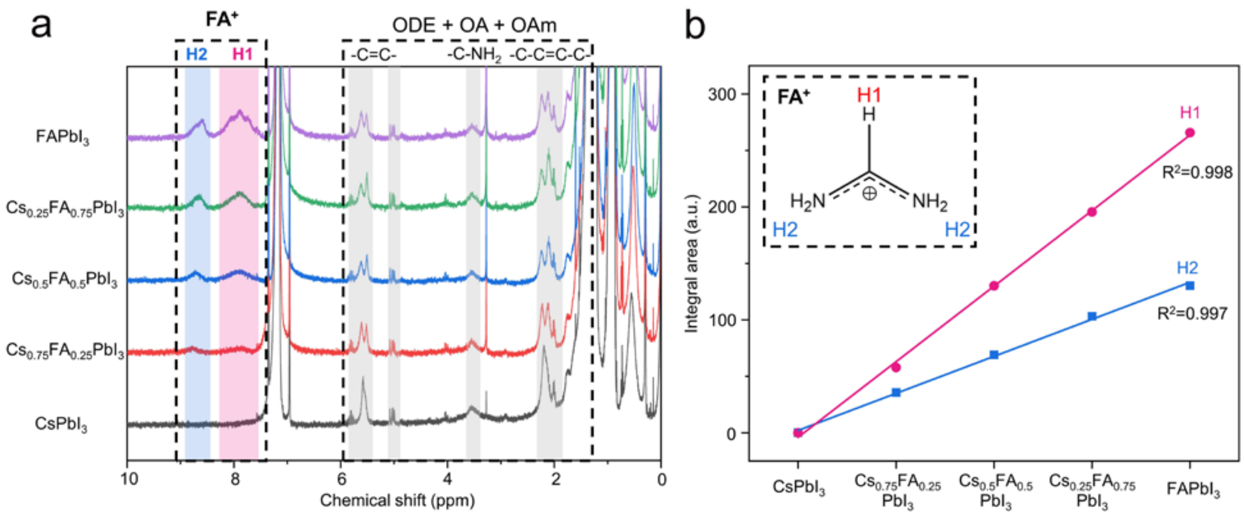

Supplementary Figure 2. Characterization of FA composition in as-synthesized $Cs_xFA_{1-x}PbI_3$ QDs. a,b 1H NMR spectra and the integral area of C-H (H1) and N-H (H2) in FA^+ cation for $Cs_xFA_{1-x}PbI_3$ PQDs with different compositions.

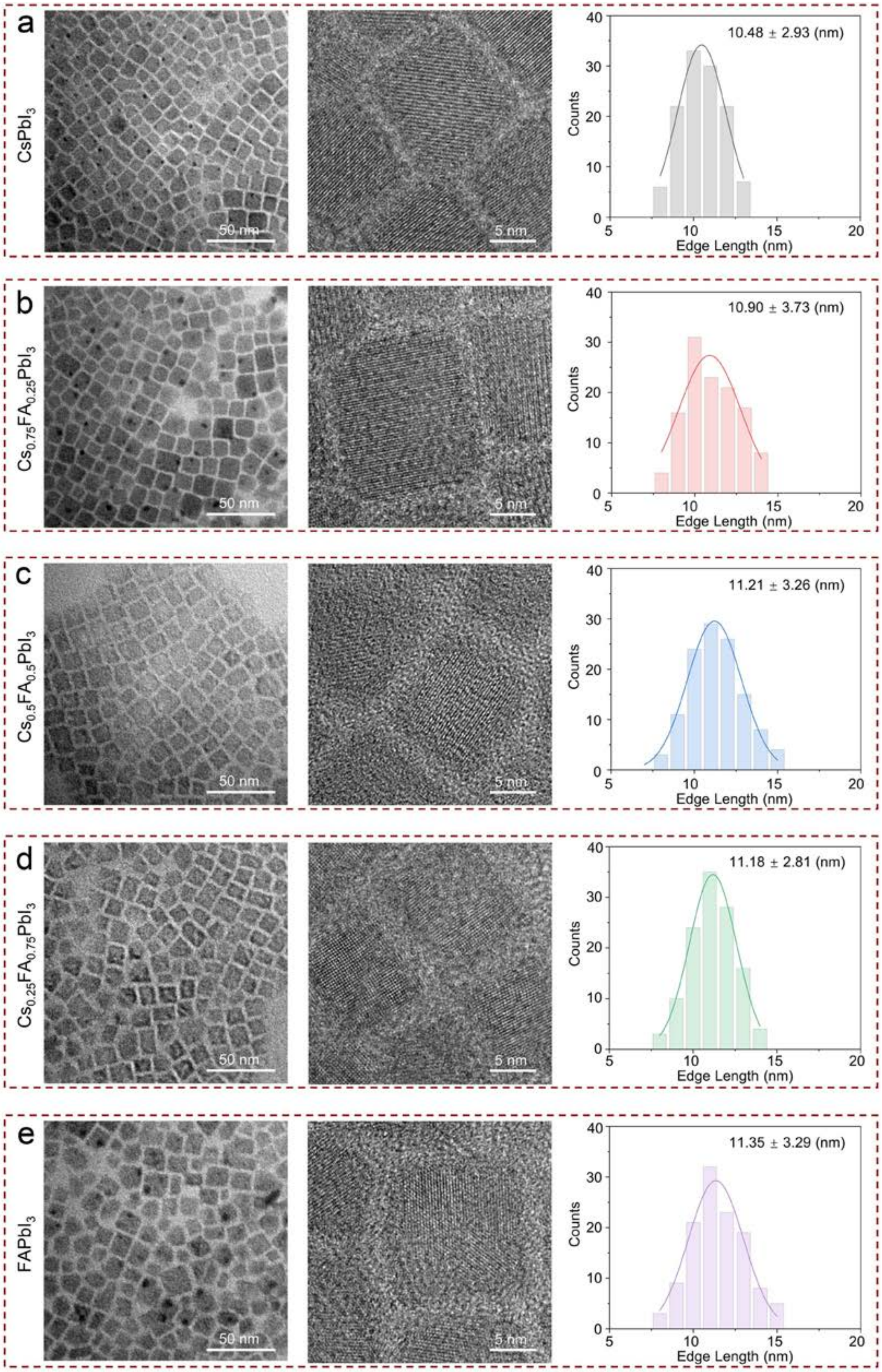

Supplementary Figure 3. Morphology of as-synthesized $Cs_xFA_{1-x}PbI_3$ QDs. a-e TEM images (left panel) and size distribution (right panel) of $CsPbI_3$, $Cs_{0.75}FA_{0.25}PbI_3$, $Cs_{0.5}FA_{0.5}PbI_3$, $Cs_{0.25}FA_{0.75}PbI_3$, and $FAPbI_3$ PQDs.

Supplementary Table 1. Parameters used in Figure 1b for XRD analyses. 2θ angles used in Figure 1b drawn from in situ XRD data for $Cs_xFA_{1-x}PbI_3$ PQDs.

	**PVK-QDs**		**PVK-bulk films**		**non-PVK yellow phase**	**PbI_2**
$FAPbI_3$	28.00°	31.17°	27.54°	30.91°	36.50°	38.05°
$Cs_{0.25}FA_{0.75}PbI_3$	28.15°	31.25°	27.68°	31.04°	36.80°	38.10°
$Cs_{0.5}FA_{0.5}PbI_3$	28.25°	31.50°	27.87°	31.15°	36.85°	38.08°
$Cs_{0.75}FA_{0.25}PbI_3$	28.37°	31.61°	28.00°	31.35°	37.10°	38.02°
$CsPbI_3$	28.55°	31.75°	28.10°	31.47°	37.20°	38.00°

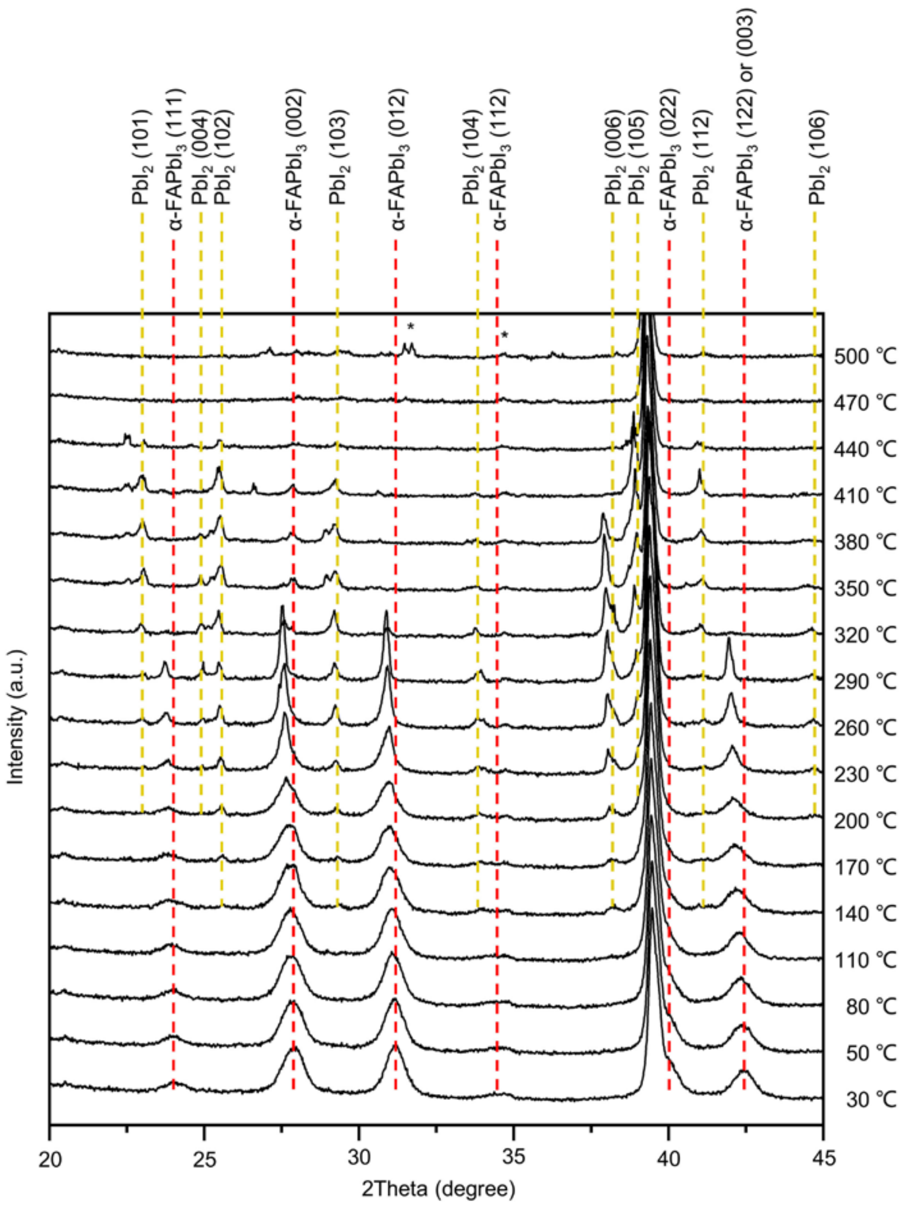

Supplementary Figure 4. Analysis details of in situ XRD profiles for $FAPbI_3$ PQDs. The data are collected at different elevated temperatures ranging from 30 °C to 500 °C. The peaks of substrate are marked with asterisks.

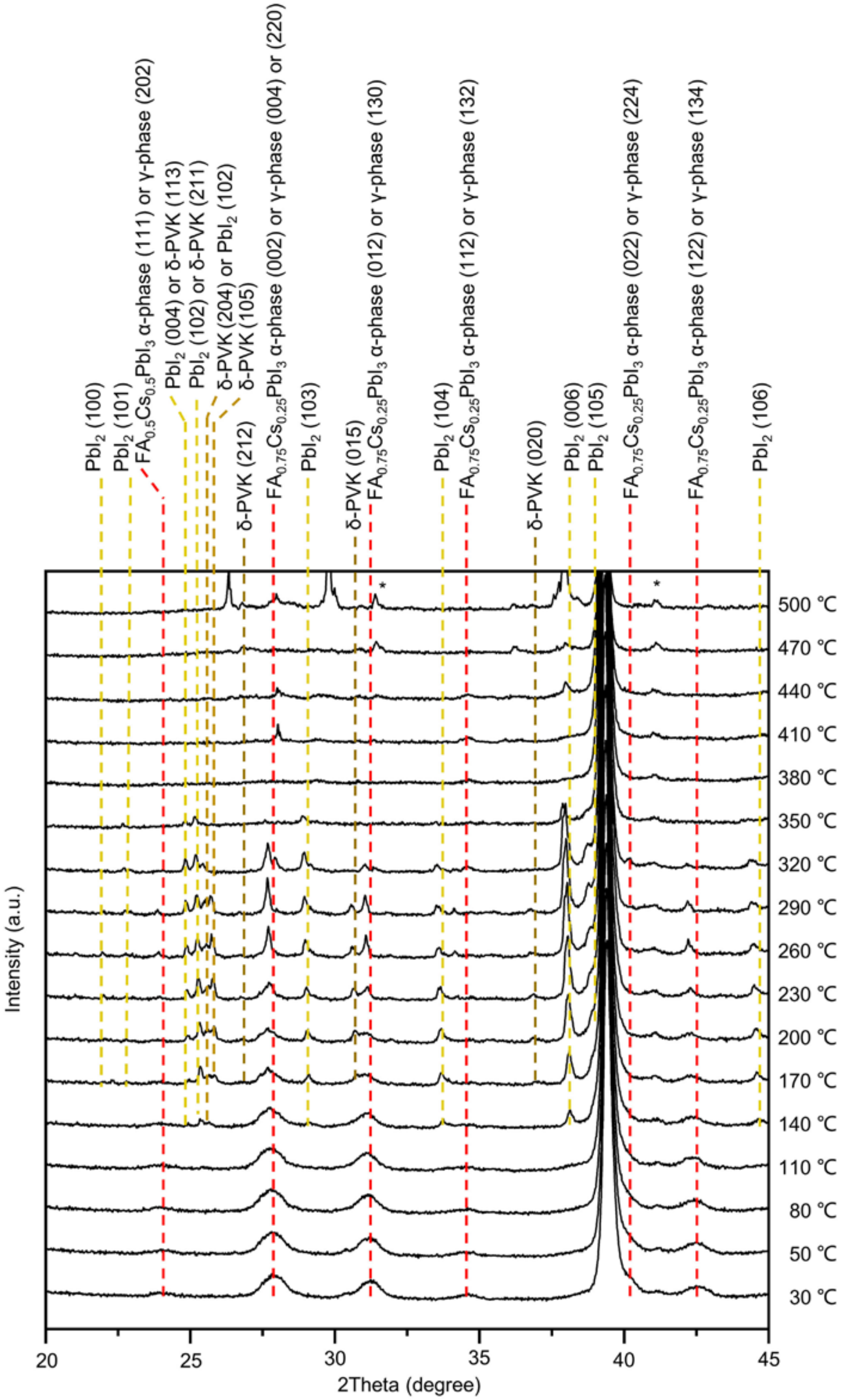

Supplementary Figure 5. Analysis details of in situ XRD profiles for $Cs_{0.25}FA_{0.75}PbI_3$ PQDs. The data are collected at different elevated temperatures ranging from 30 °C to 500 °C. The peaks of substrate are marked with asterisks.

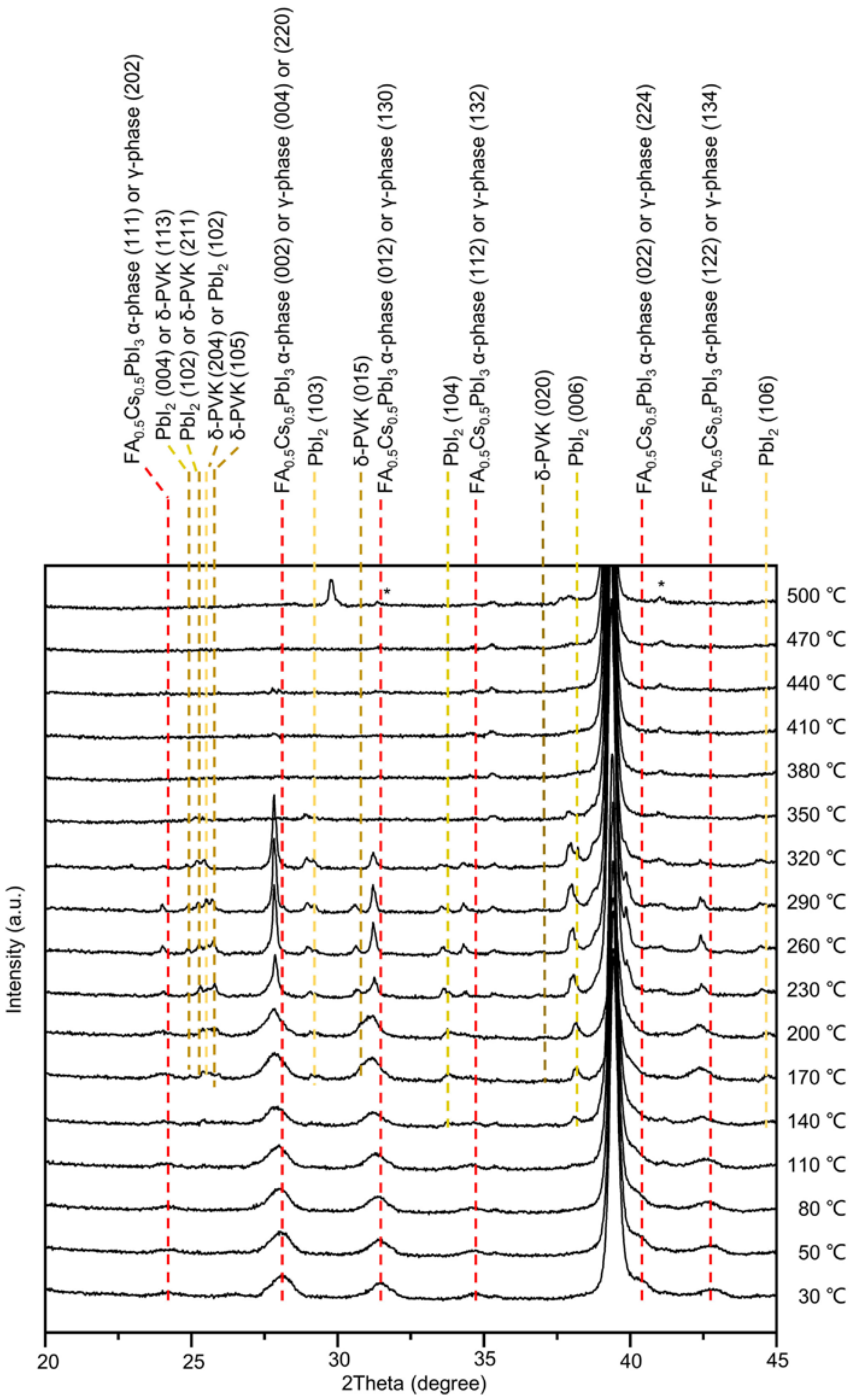

Supplementary Figure 6. Analysis details of in situ XRD profiles for $Cs_{0.5}FA_{0.5}PbI_3$ PQDs. The data are collected at different elevated temperatures ranging from 30 °C to 500 °C. The peaks of substrate are marked with asterisks.

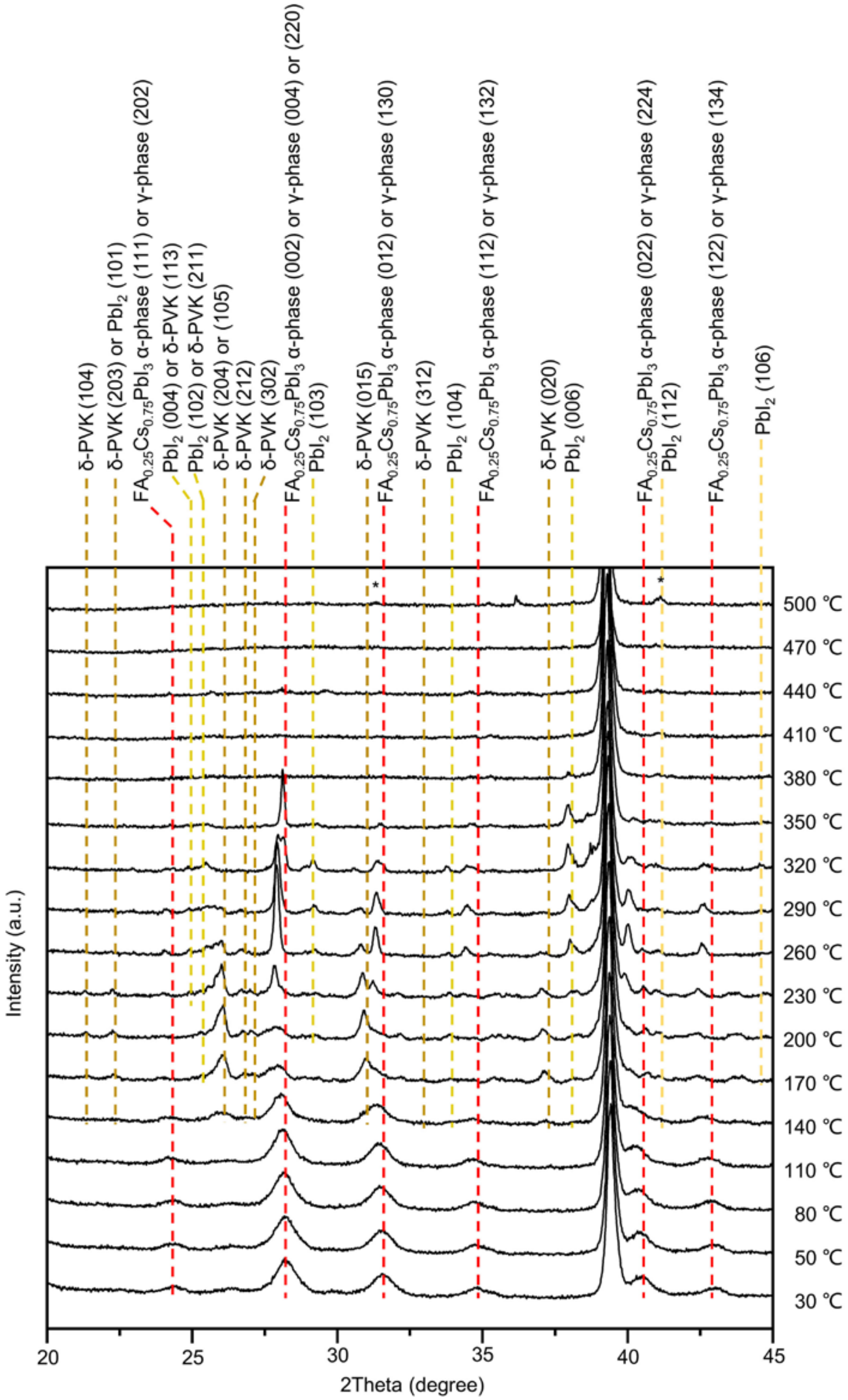

Supplementary Figure 7. Analysis details of in situ XRD profiles for $Cs_{0.75}FA_{0.25}PbI_3$ PQDs. The data are collected at different elevated temperatures ranging from 30 °C to 500 °C. The peaks of substrate are marked with asterisks.

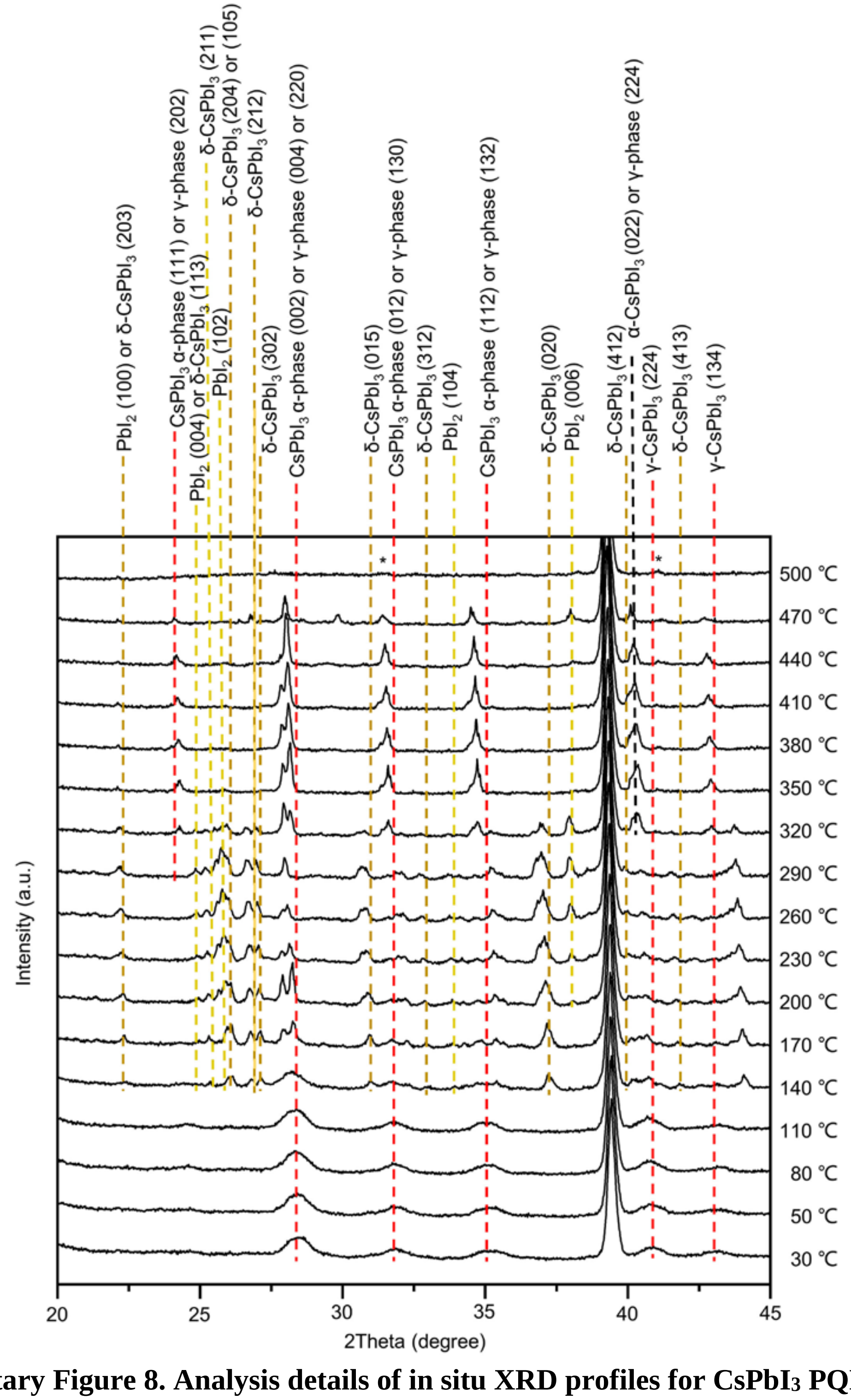

Supplementary Figure 8. Analysis details of in situ XRD profiles for $CsPbI_3$ PQDs. The data are collected at different elevated temperatures ranging from 30 °C to 500 °C. The peaks of substrate are marked with asterisks.

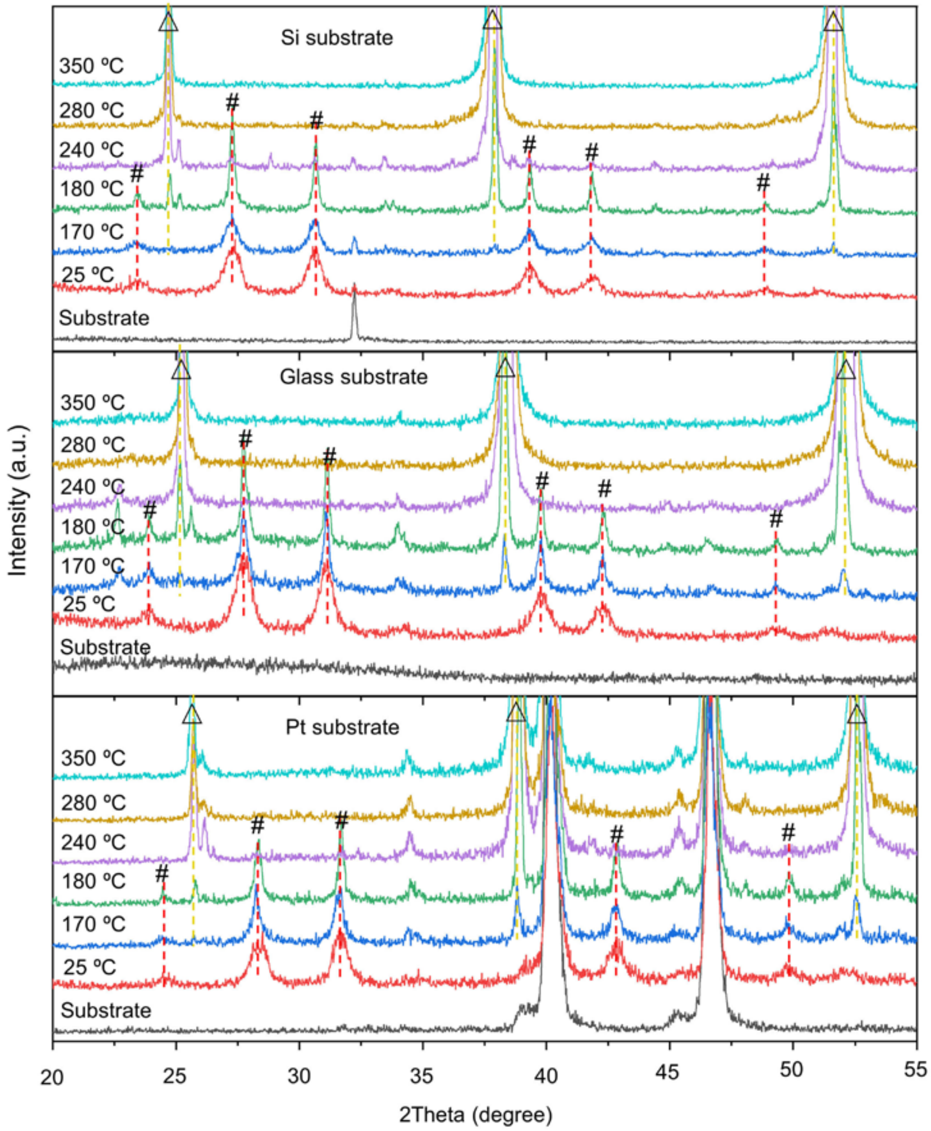

Supplementary Figure 9. Influence of the substrates used for XRD measurements. The XRD profiles of $FAPbI_3$ PQDs on different substrates are collected at different elevated temperatures. The peaks of PbI_2 and black-phase PQDs are marked with Δ and #, respectively.

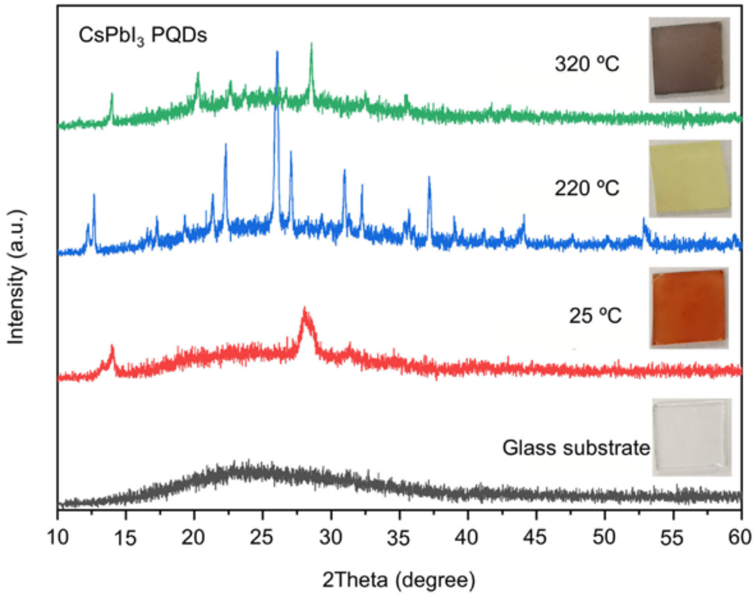

Supplementary Figure 10. Phase transition of $CsPbI_3$ PQDs during heating. The XRD profiles of $CsPbI_3$ PQDs are collected at 25°C, 190°C and 330°C. The insets (right) are the corresponding photographs of $CsPbI_3$ samples at different phase transition stages.

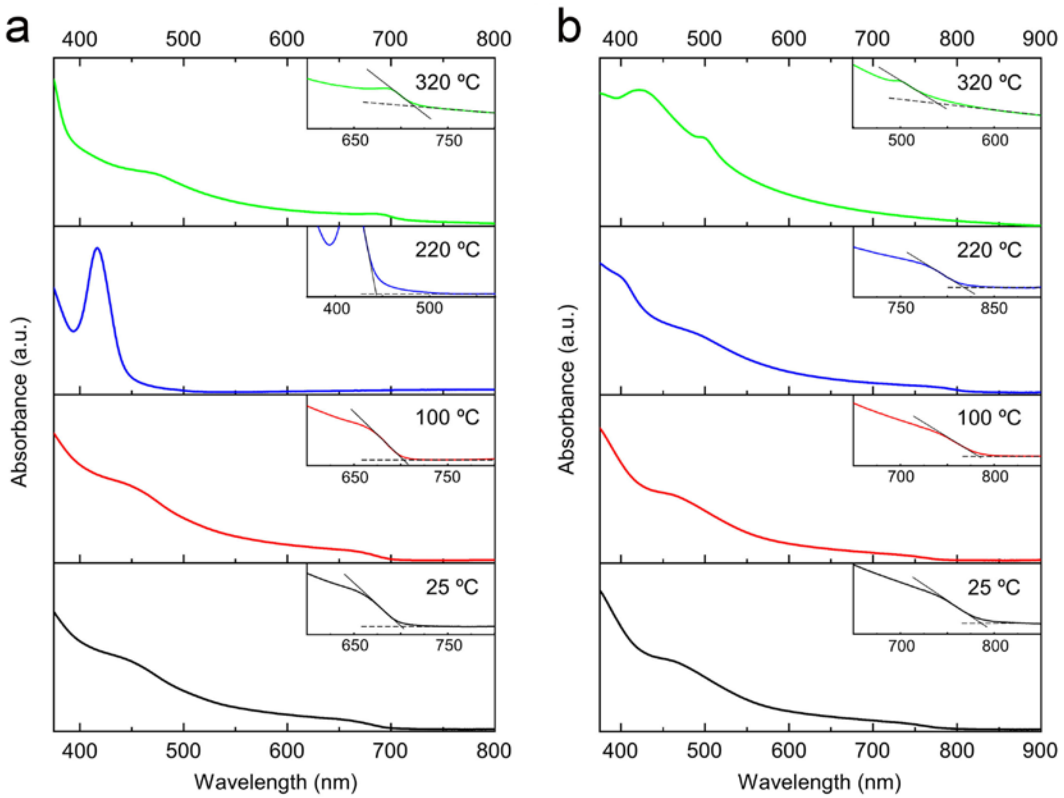

Supplementary Figure 11. Changes of absorption properties for $CsPbI_3$ and $FAPbI_3$ PQDs during heating. a,b UV-Vis spectra of pure $CsPbI_3$ and $FAPbI_3$ PQDs are recorded at 25°C, 100°C, 220°C. and 320°C.

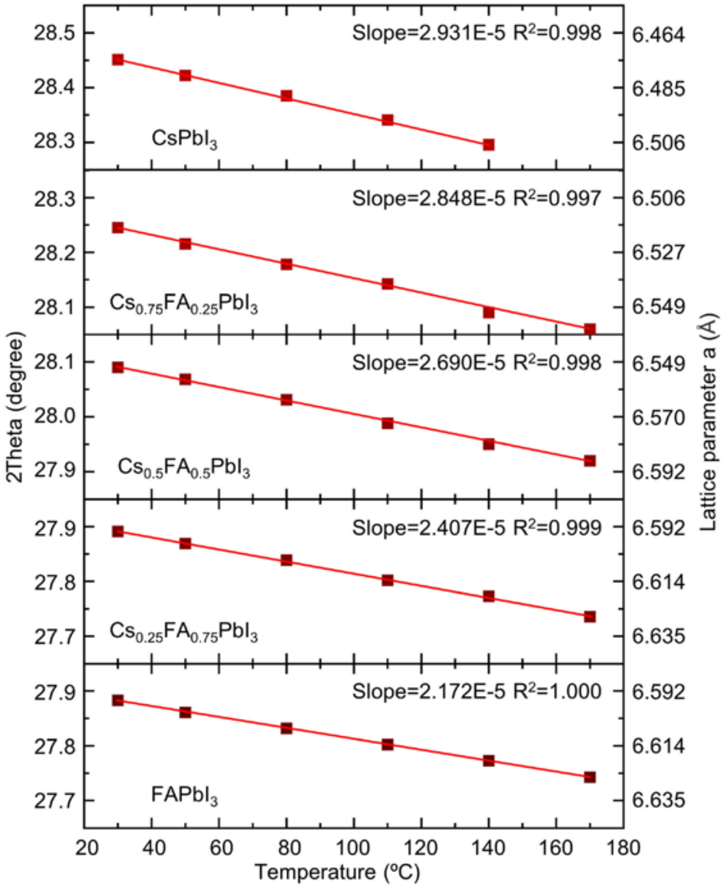

Supplementary Figure 12. Lattice thermal expansion of $Cs_xFA_{1-x}PbI_3$ QDs. The shifts of the perovskite (002) peak (for cubic phase) with increasing temperature are plotted for $FAPbI_3$, $Cs_{0.25}FA_{0.75}PbI_3$, $Cs_{0.5}FA_{0.5}PbI_3$, $Cs_{0.75}FA_{0.25}PbI_3$, and $CsPbI_3$ PQDs

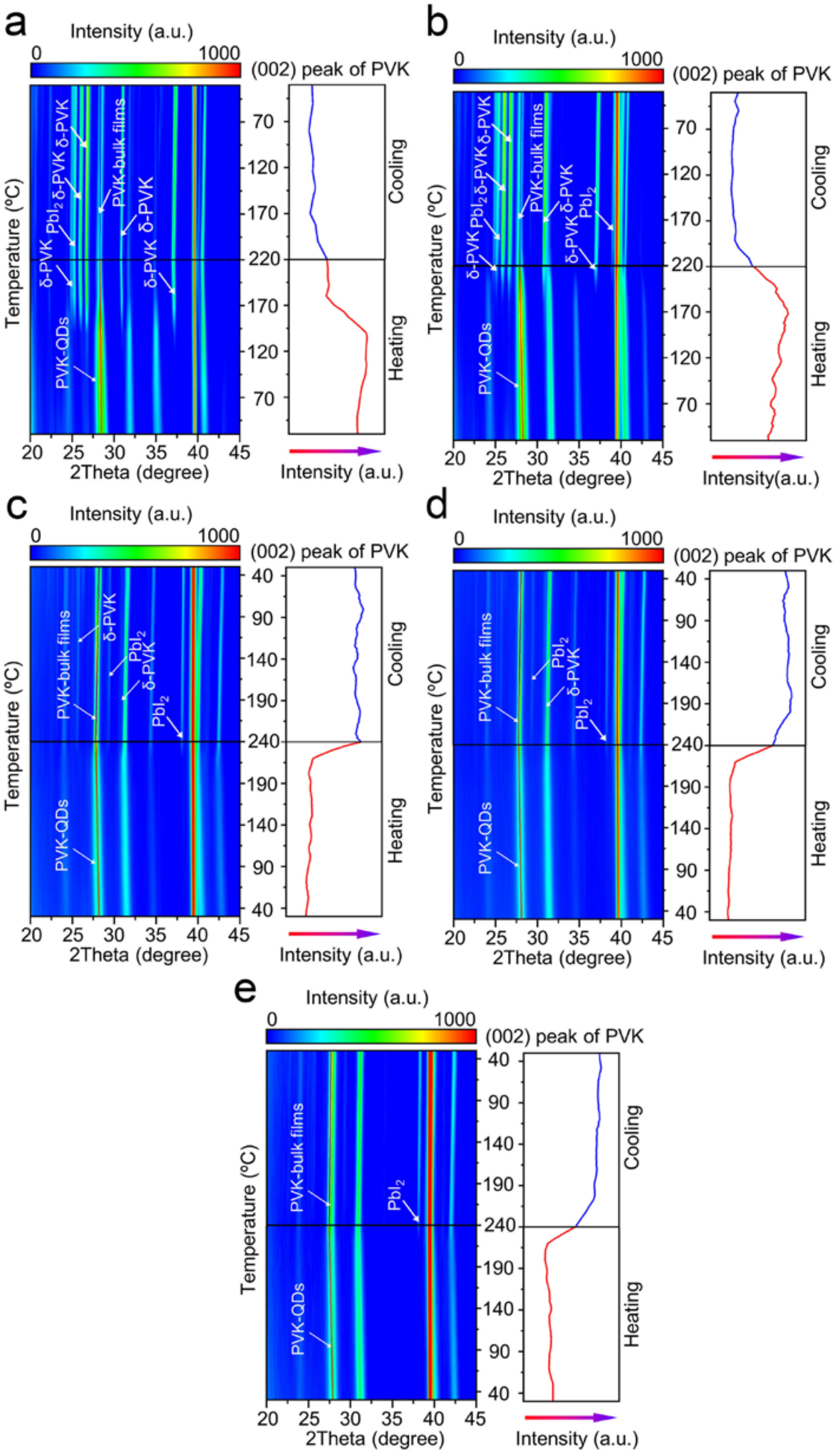

Supplementary Figure 13. In situ XRD experiments for $Cs_xFA_{1-x}PbI_3$ QDs during one heating-cooling cycle. a-e The data are collected for $CsPbI_3$, $Cs_{0.75}FA_{0.25}PbI_3$, $Cs_{0.5}FA_{0.5}PbI_3$, $Cs_{0.25}FA_{0.75}PbI_3$, and $FAPbI_3$ PQDs as the temperature is increased up to phase transition or decomposition temperature and then cooled down to room temperature.

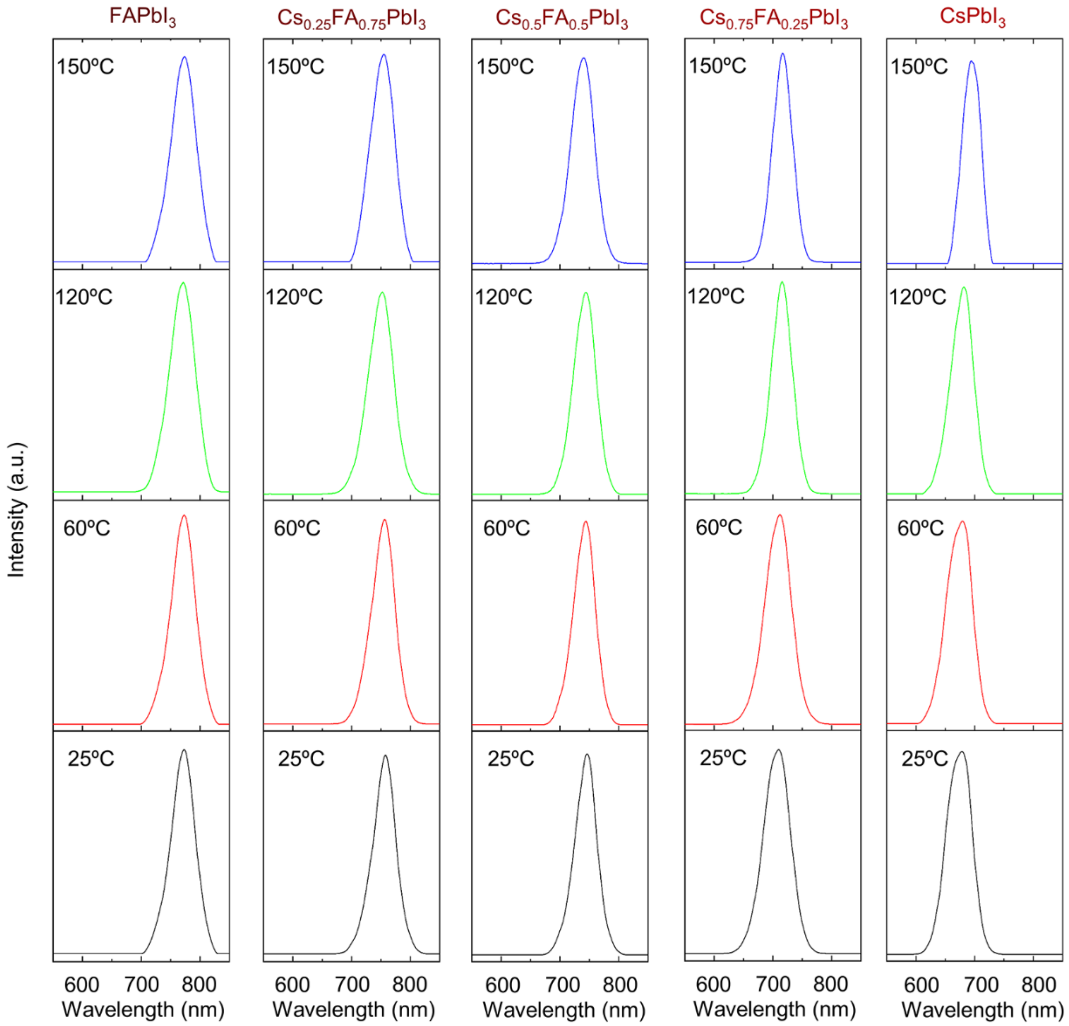

Supplementary Figure 14. PL stability of $Cs_xFA_{1-x}PbI_3$ QDs. The PL spectra of $FAPbI_3$, $Cs_{0.25}FA_{0.75}PbI_3$, $Cs_{0.5}FA_{0.5}PbI_3$, $Cs_{0.75}FA_{0.25}PbI_3$, and $CsPbI_3$ PQDs are recorded when heated at 25°C, 60°C, 120°C and 150°C.

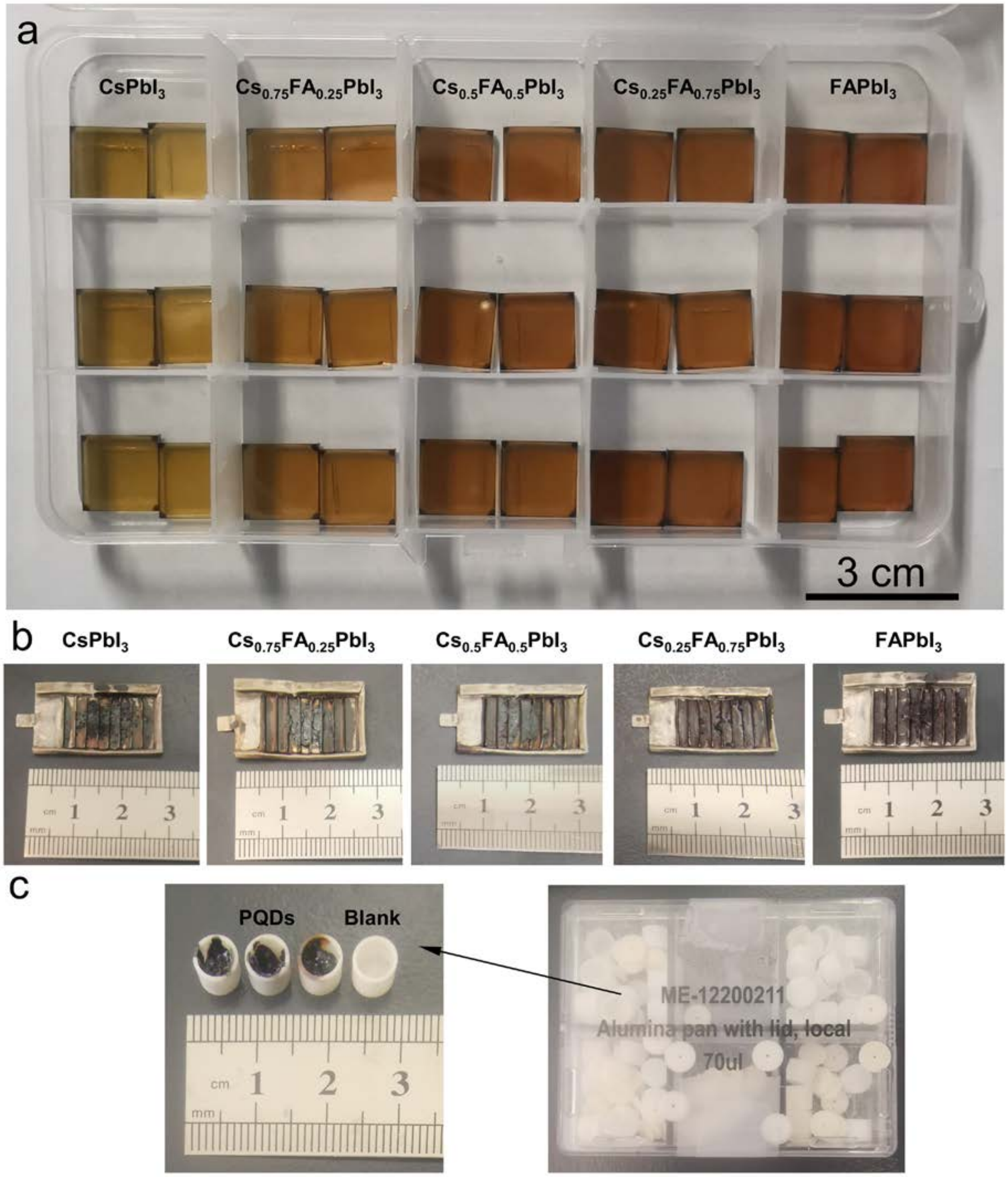

Supplementary Figure 15. The QD samples used in different characterizations. a-c Photographs of PQD samples used in SEM, in situ XRD, and TGA measurements with their corresponding holders or substrates.

Supplementary Table 2. Parameters obtained from DFT calculations for $Cs_xFA_{1-x}PbI_3$ QDs. The values of ligand binding energy and formation energy are calculated for $FAPbI_3$, $Cs_{0.25}FA_{0.75}PbI_3$, $Cs_{0.5}FA_{0.5}PbI_3$, $Cs_{0.75}FA_{0.25}PbI_3$, and $CsPbI_3$ PQDs with OA or OAm ligands.

**PQD with ligands**	**Binding energy (eV)**		**Formation energy (eV/Pb atom)**	
	OA	OAm	OA	OAm
$FAPbI_3$	-4.18	-4.50	-66.06	-66.24
$Cs_{0.25}FA_{0.75}PbI_3$	-2.58	-2.27	-47.34	-47.45
$Cs_{0.5}FA_{0.5}PbI_3$	-1.54	-1.38	-40.19	-40.31
$Cs_{0.75}FA_{0.25}PbI_3$	-0.55	-0.54	-32.94	-33.06
$CsPbI_3$	-0.41	-0.47	-30.26	-30.42

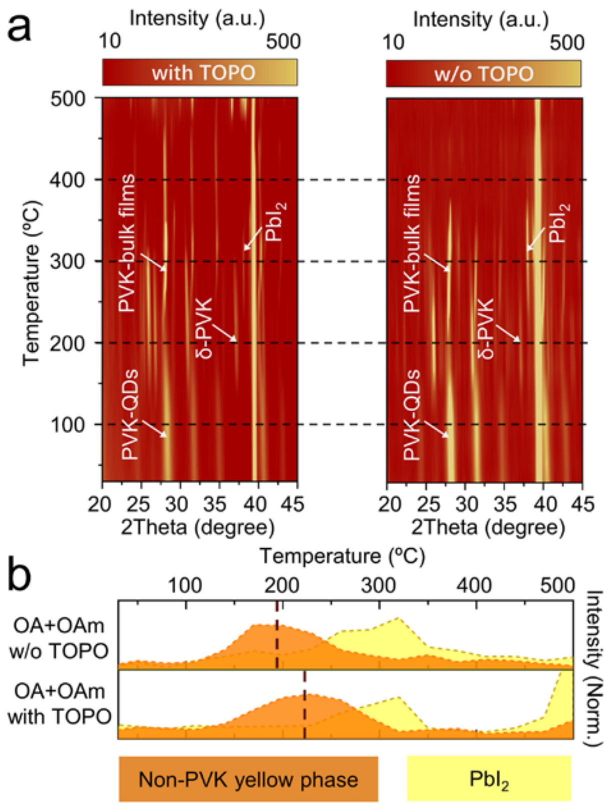

Supplementary Figure 16. In situ XRD experiments for $Cs_{0.75}FA_{0.25}PbI_3$ PQDs with TOPO ligand capping. a In situ XRD patterns collected from 30 °C to 500 °C under argon flowing for $Cs_{0.75}FA_{0.25}PbI_3$ PQDs with (left panel) and without (right panel) TOPO ligands. **b** Scheme for the dominant reflections in the thermal degradation of $Cs_{0.75}FA_{0.25}PbI_3$ PQDs with (below) and without (upper) TOPO ligands that is drawn from the vertical line cuts of the 2-dimensional in situ XRD patterns taken at the 2θ angles shown in Supplementary Table 1 for orthorhombic non-perovskite (non-PVK yellow phase) and PbI_2.

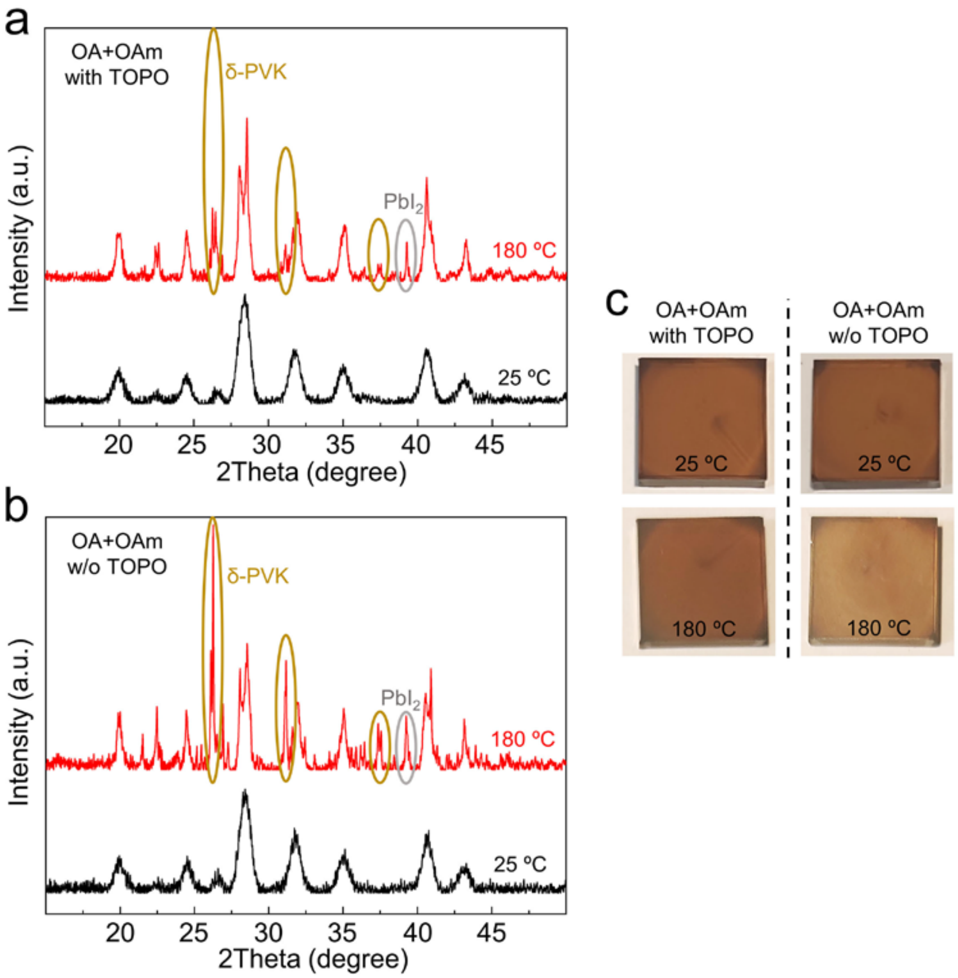

Supplementary Figure 17. Comparison of thermal stability between $Cs_{0.75}FA_{0.25}PbI_3$ PQDs with and without TOPO ligands. a,b XRD patterns of $Cs_{0.75}FA_{0.25}PbI_3$ PQDs with and without TOPO ligands are obtained at 25 °C and 180 °C. **c** Photographs of $Cs_{0.75}FA_{0.25}PbI_3$ PQD films with and without TOPO ligands heated at different temperatures.

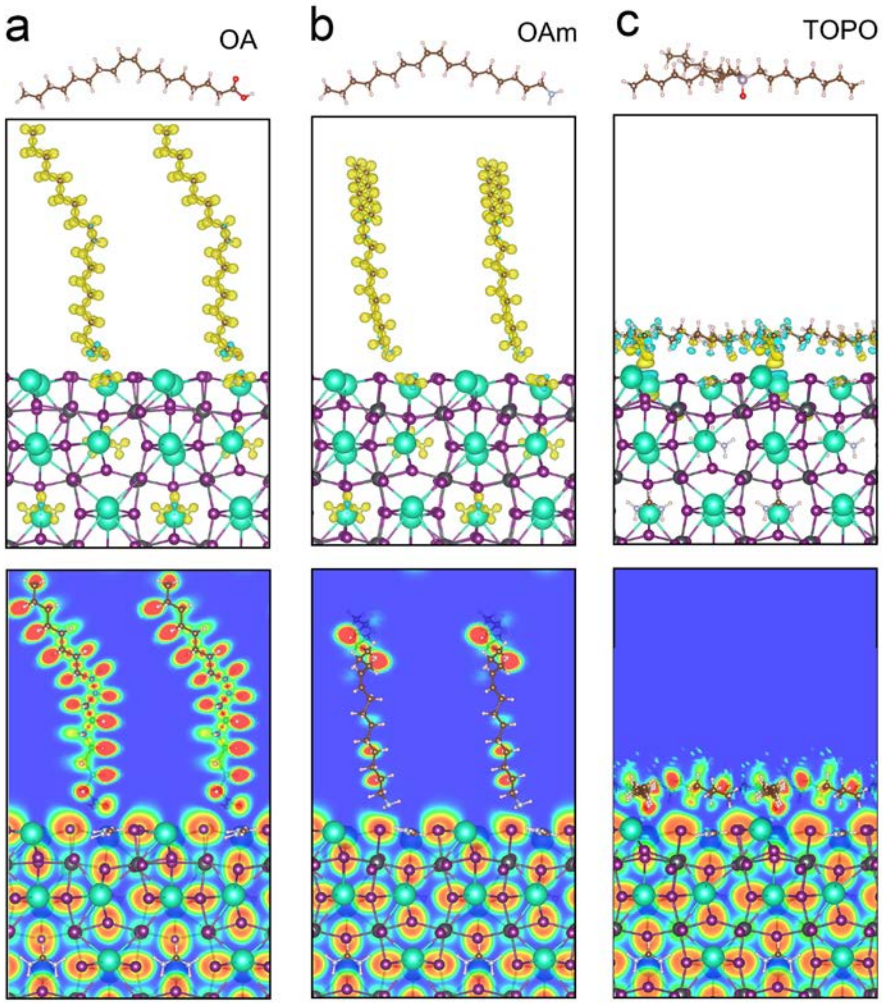

Supplementary Figure 18. Comparison of ligand binding properties between $Cs_{0.75}FA_{0.25}PbI_3$ PQDs with OA, OAm and TOPO ligands. a-c Charge density difference plots (upper) and electron localization function analyses (below) for OA, OAm or TOPO ligands adsorbed on the surface of $Cs_xFA_{1-x}PbI_3$ PQDs. Red and green denotes areas of electron accumulation and depletion in electron localization function analyses, respectively.

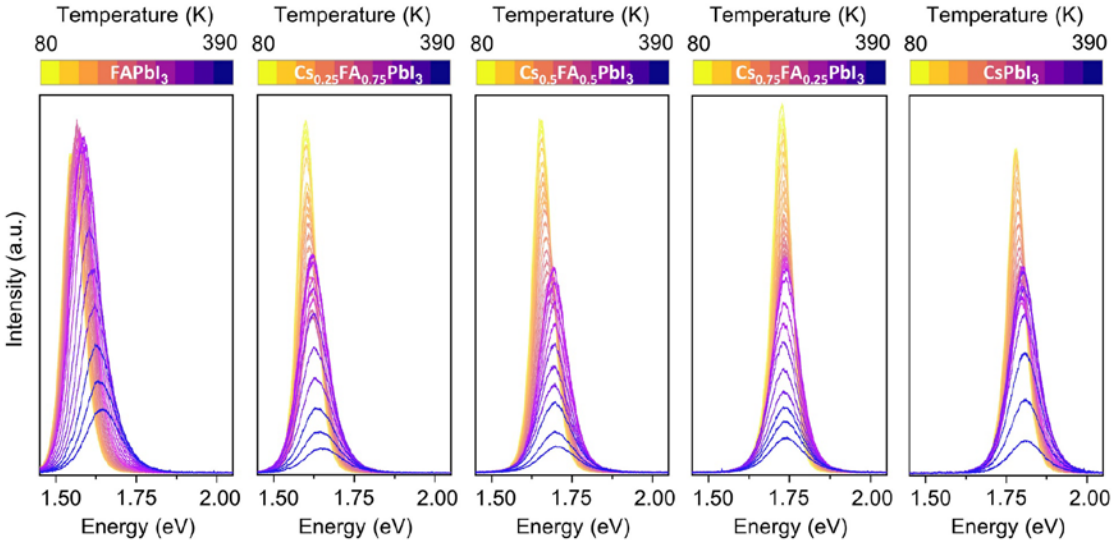

Supplementary Figure 19. 1D plots of in situ PL spectra for $Cs_xFA_{1-x}PbI_3$ QDs. The data are collected from 80 K to 390 K for $FAPbI_3$, $Cs_{0.25}FA_{0.75}PbI_3$, $Cs_{0.5}FA_{0.5}PbI_3$, $Cs_{0.75}FA_{0.25}PbI_3$, and $CsPbI_3$ PQDs.

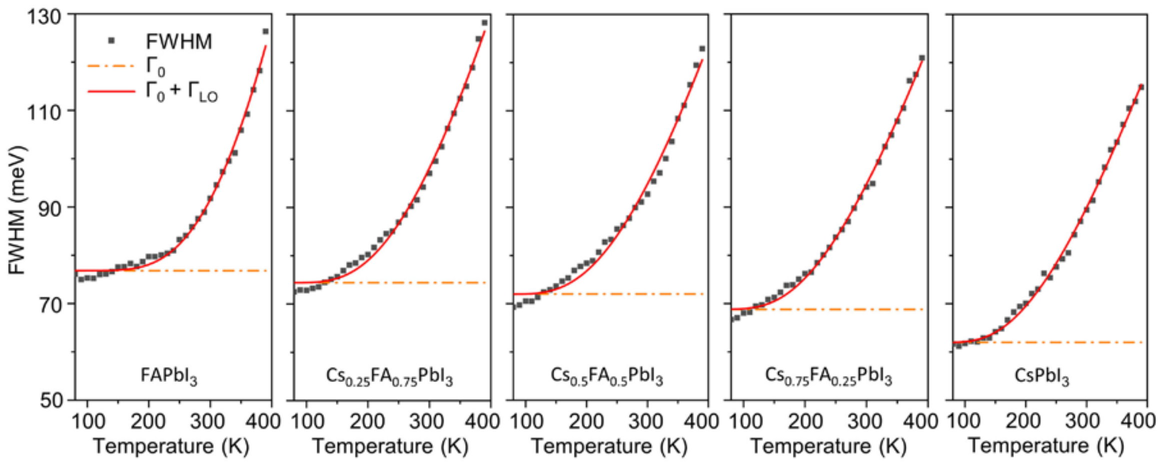

Supplementary Figure 20. FWHM extracted from in situ PL spectra of $Cs_xFA_{1-x}PbI_3$ QDs fitted only with Γ_0 and Γ_{LO}. The black squares are the experimental data fitted using Segall's expression (red solid line) with longitudinal optical phonon interaction $\Gamma(T) = \Gamma_0 + \Gamma_{LO}$. The orange dash-dot line represents the temperature dependence of the PL exciton linewidth broadening arising from inhomogeneous broadening term $\Gamma(T) = \Gamma_0$.

Supplementary Table 3. Parameters obtained from the fittings in Supplementary Figure 20. Extracted parameters are calculated by Eq.4 only including Γ_0 and Γ_{LO} terms for $FAPbI_3$, $Cs_{0.25}FA_{0.75}PbI_3$, $Cs_{0.5}FA_{0.5}PbI_3$, $Cs_{0.75}FA_{0.25}PbI_3$, and $CsPbI_3$ PQDs. The noted uncertainties are the standard-errors from the fit procedures without including the systematic errors from the performed experiments.

PQDs	Γ_0 (meV)	E_{LO} (meV)	γ_{LO} (meV)
$CsPbI_3$	62.0±0.35	64.6±2.08	311.6±22.8
$Cs_{0.75}FA_{0.25}PbI_3$	68.8±0.38	69.4±2.54	352.8±30.9
$Cs_{0.5}FA_{0.5}PbI_3$	72.0±0.57	78.7±4.55	455.2±69.7
$Cs_{0.25}FA_{0.75}PbI_3$	74.4±0.42	83.0±3.31	561.2±62.0
$FAPbI_3$	76.8±0.31	127.1±4.60	1984.1±286.6

Supplementary Table 4. Parameters obtained from the fittings in Figure 5b. Extracted parameters are calculated by Eq.4 only including Γ_0, Γ_{AC} and Γ_{LO} terms for $FAPbI_3$, $Cs_{0.25}FA_{0.75}PbI_3$, $Cs_{0.5}FA_{0.5}PbI_3$, $Cs_{0.75}FA_{0.25}PbI_3$, and $CsPbI_3$ PQDs. The noted uncertainties are the standard-errors from the fit procedures without including the systematic errors from the performed experiments

**PQDs**	Γ_0 **(meV)**	γ_{AC} **(meV)**	E_{LO} **(meV)**	γ_{LO} **(meV)**
$CsPbI_3$	60.0±0.31	0.019	70.9±2.26	349.0±27.1
$Cs_{0.75}FA_{0.25}PbI_3$	62.7±0.18	0.055	98.4±2.44	652.4±51.7
$Cs_{0.5}FA_{0.5}PbI_3$	66.6±0.32	0.046	104.6±5.67	764.9±145.9
$Cs_{0.25}FA_{0.75}PbI_3$	69.5±0.22	0.042	109.4±2.97	1036.5±98.5
$FAPbI_3$	74.9±0.24	0.014	141.2±4.31	2837.4±383.6

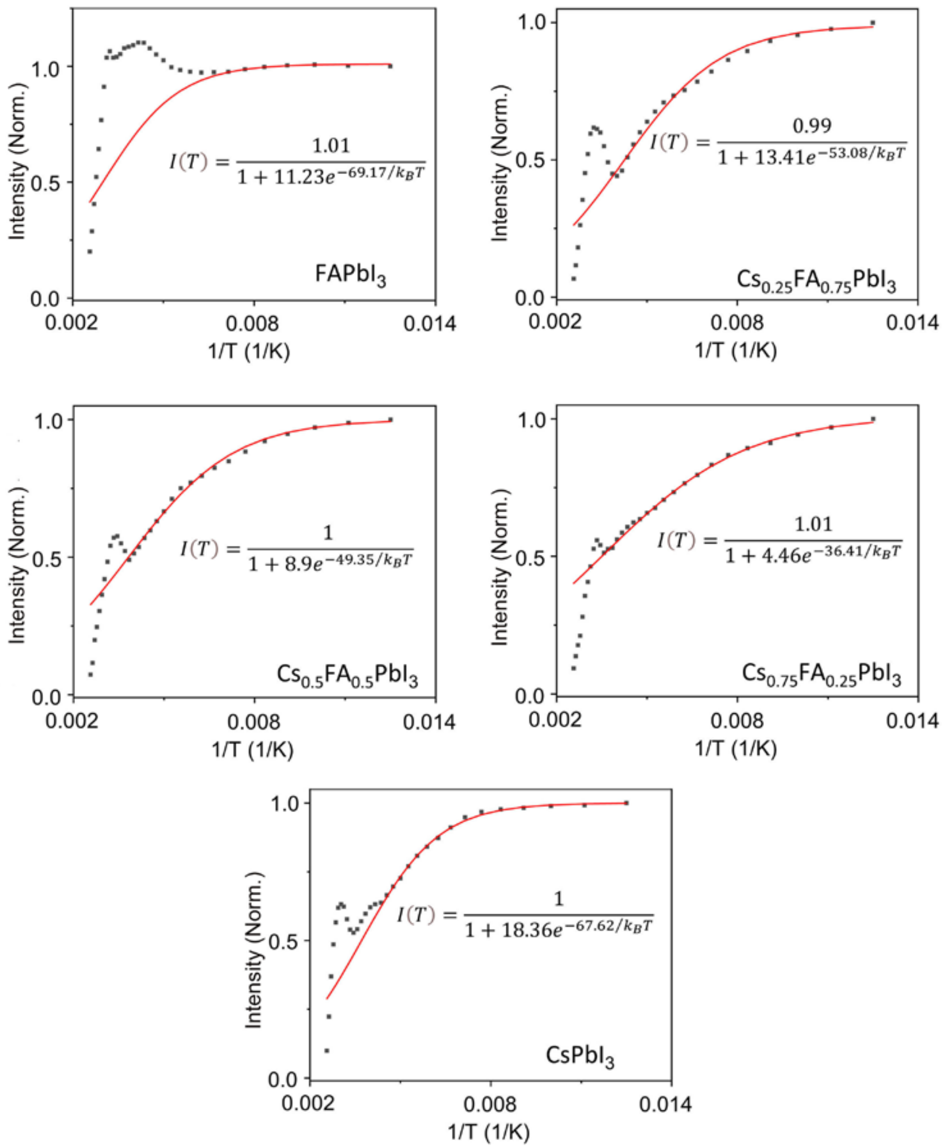

Supplementary Figure 21. Details on the temperature dependence of PL intensity for $Cs_xFA_{1-x}PbI_3$ PQDs. The normalized PL intensity data are extracted from in situ PL spectra of different $Cs_xFA_{1-x}PbI_3$ PQDs.

Supplementary Table 5. Comparison of exciton binding energies between different reports. The values of exciton binding energy, $E_{b,ex}$, are derived from the previous reports and this work.

**Perovskite materials**	**$E_{b,ex}$ (meV)**	**Reference**
$CsPbI_3$ QDs	55	*Angew. Chem. Int. Ed.* **2020**, 59, 22230-22237.
$CsPbI_3$ QDs	25	*Adv. Funct. Mater.* **2018**, 28, e1800945.
$CsPbI_3$ QDs	45	*J. Phys. Chem. C* **2017**, 121, 26054-26062.
$CsPbI_3$ bulks	103	*J. Mater. Chem. A* **2019**, 7, 22539-22549.
$CsPbI_3$ QDs	59.6	*Adv. Mater.* **2021**, 33, e2008820.
$FAPbI_3$ bulks	48.9	*Opt. Mater.* **2021**, 121, 111586.
$FAPbI_3$ bulks	91	*Light-Sci. Appl.* **2016**, 5, e16056.
$CsPbI_3$ QDs	67.2	This work
$Cs_{0.75}FA_{0.25}PbI_3$ QDs	36.4	This work
$Cs_{0.5}FA_{0.5}PbI_3$ QDs	49.4	This work
$Cs_{0.25}FA_{0.75}PbI_3$ QDs	53.1	This work
$FAPbI_3$ QDs	69.2	This work
